# Trends in Incident Dementia Diagnosis Before and After Medicare Risk Adjustment

**DOI:** 10.1001/jamanetworkopen.2023.47708

**Published:** 2023-12-15

**Authors:** Julie M. Zissimopoulos, Geoffrey F. Joyce, Mireille Jacobson

**Affiliations:** 1 USC Price School of Public Policy, University of Southern California, Los Angeles; 2 Leonard D. Schaeffer Center for Health Policy and Economics, University of Southern California, Los Angeles; 3 Leonard Davis School of Gerontology, University of Southern California, Los Angeles; 4 USC School of Pharmacy, University of Southern California, Los Angeles

## Abstract

This cohort study examines rates of new diagnosis of Alzheimer disease and related dementias among beneficiaries of Medicare Advantage plans vs traditional Medicare from 2016 through 2020.

## Introduction

Medicare reintroduced risk adjustment for Alzheimer disease and related dementias (ADRD) to its Medicare Advantage (MA) plan payments in 2020, which provides incentive to improve dementia detection and reduce missed diagnoses but also renews concerns about diagnostic upcoding^1^ for over 30 million MA beneficiaries. We assessed annual trends in incident ADRD diagnosis for all beneficiaries in MA and compared them with trends in traditional Medicare (TM) from 2016 through 2020.

## Methods

This cohort study used 2015-2020 encounter data for 100% of MA enrollees and claims for 100% of TM beneficiaries. Beneficiaries were 66 years or older and continuously enrolled in TM (with Part D) or MA for at least 2 years. In accordance with the Common Rule, this study was exempt from review and informed consent because it was not human participant research. We followed the STROBE reporting guideline.

Sensitivity analysis allowed switching between MA and TM. Main outcome was the annual percentage of previously dementia-free MA and TM enrollees with an incident dementia diagnosis, adjusted for age, sex, and race and ethnicity (coded using improved classification of self-reported Social Security Administration data^2^) to match 2016 cohorts. Diagnosis was defined using Hierarchical Condition Category codes from *International Classification of Diseases, Ninth Revision* and *International Statistical Classification of Diseases and Related Health Problems, Tenth Revision* (eTable in Supplement 1). The ADRD risk adjustment was announced in 2018 and applied to 2019 claims.

We estimated covariate-adjusted ordinary least-squares regressions and reported Chow test *F* statistic for structural break in 2019. Two-sided *P* < .001 indicated significance. Analyses were performed between August and October 2023 using SAS Enterprise Guide 7.1 (SAS Institute, Inc).

## Results

In 2016, 11 936 912 MA (57.4% females, 42.6% males; mean [SD] age, 74.7 [6.8] years) and 13 983 621 TM (75.2% females, 24.8% males; mean [SD] age, 75.2 [7.2] years) beneficiaries met inclusion criteria. The percentage of MA beneficiaries with incident dementia decreased from 3.5% in 2016 to 3.4% in 2018 before reversing in 2019 (Figure). In 2019, 3.7% of beneficiaries had incident dementia diagnosis, a 0.27–percentage point increase or 7.8% relative increase from 2018. Null hypothesis of no break in trend in 2019 was rejected based on *F* statistic (*F* = 393; *P* < .001). New diagnoses increased by 32 000 persons between 2017 and 2018 but by over 74 000 between 2018 and 2019. In 2020, the percentage of MA beneficiaries with incident dementia diagnosis was 3.6%. Percentage of TM beneficiaries with incident dementia diagnosis decreased every year from 4.3% in 2016 to 3.9% in 2019 and 3.6% in 2020.

**Figure.**
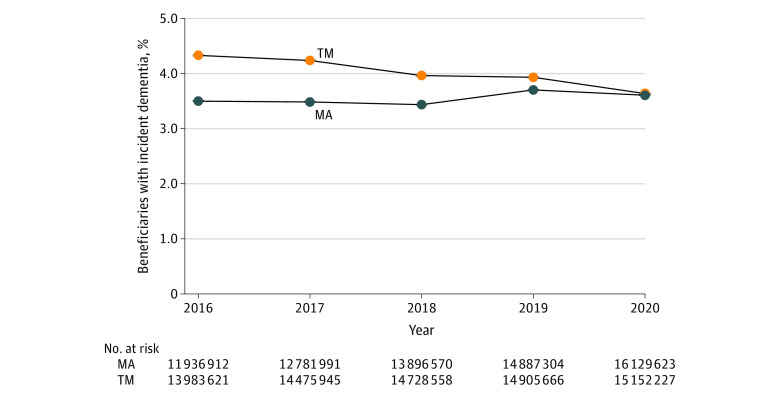
Incident Dementia Diagnosis Among Medicare Advantage (MA) and Traditional Medicare (TM) Beneficiaries

## Discussion

Beginning in 2019, ADRD diagnosis was used to adjust MA plan payments. The increase in diagnoses among MA beneficiaries contrasted with a continued decrease among TM beneficiaries as well as with findings of decreasing ADRD incidence in the US and Western Europe potentially associated with improved cardiovascular health and/or social and educational experiences.^3^ Dementia diagnosis rates were lower among MA than TM beneficiaries,^4^ which may reflect differences in care access, delivery, and financing.^5^ The ADRD risk adjustment decreased MA and TM disparities and may reflect improved dementia detection with better financial and care planning. However, it may reflect inappropriate upcoding and overdiagnosis. Evidence is needed to distinguish between these possibilities.

This study has limitations. Data were available only through 2020, and 2020 diagnoses rates partially reflected reduced health care use due to COVID-19. Differential selection associated with increases in switching from TM to MA over time^6^ may affect trends, although estimates of sample inclusion of switchers were robust. If increases in dementia diagnosis capture improved dementia detection, financial incentives may be leveraged to improve care quality for persons with a diagnosis.

## Notes

Supplement 1. **eTable.** ICD Codes

Supplement 2.
Data Sharing Statement
