# Supplementary material for: Trends in Incident Dementia Diagnosis Before and After Medicare Risk Adjustment
Source: JAMA Netw Open. 2023 Dec 15;6(12):e2347708. doi: 10.1001/jamanetworkopen.2023.47708 (PMC10724756; doi:10.1001/jamanetworkopen.2023.47708)
Supplement: Supplement 1. — eTable. ICD Codes [file jamanetwopen-e2347708-s001.pdf]

Supplemental Online Content

Zissimopoulos JM, Joyce GF, Jacobson M. Trends in incident dementia diagnosis before and after Medicare risk adjustment. *JAMA Netw Open*. 2023;6(12):e2347708. doi:10.1001/jamanetworkopen.2023.47708

eTable. ICD Codes

This supplemental material has been provided by the authors to give readers additional information about their work.

© 2023 Zissimopoulos J et al. *JAMA Network Open*

eTable. ICD Codes

Code Type	Code	Description
**ICD-9**	290.0	Senile dementia, uncomplicated
	290.10	Presenile dementia, uncomplicated
	290.11	Presenile dementia w/ delirium
	290.12	Presenile dementia w/ delusional features
	290.13	Presenile dementia w/ depressive features
	290.20	Senile dementia w/ delusional features
	290.21	Senile dementia w/ depressive features
	290.3	Senile dementia w/ delirium
	290.40	Vascular dementia, uncomplicated
	290.41	Vascular dementia, w/ delirium
	290.42	Vascular dementia, w/ delusions
	290.43	Vascular dementia, w/ depressed mood
	294.0	Amnestic disorder in conditions cls-elsew
	294.10	Dementia in conditions cls-elsew w/out behav disturbnc
	294.11	Dementia in conditions cls-elsew w/ behav disturbnc
	294.20	Dementia, unspecified, w/out behav disturbnc
	294.21	Dementia, unspecified, w/ behav disturbnc
	294.8	Oth persistent mental disorders due to conds cls-elsew
	331.0	Alzheimer's Disease
	331.11	Picks disease
	331.19	Other frontotemporal dementia
	331.2	Senile degeneration of brain
	331.7	Cerebral degeneration in disease cls-elsew
	797	Senility w/out mention of psychosis
	331.82	Lewy body dementia
**ICD-10**	F01.50	Vascular dementia, w/out behav disturbnc
	F01.51	Vascular dementia, w/ behav disturbnc
	F02.80	Dementia in conditions cls-elsew w/out behav disturbnc
	F02.81	Dementia in conditions cls-elsew, w/ behav disturbnc
	F03.90	Dementia, unspecified, w/out behav disturbnc
	F03.91	Dementia, unspecified, w/ behav disturbnc
	F05	Delirium due to known physiological cond
	G13.8	Systemic atrophy aff cnsl in oth diseases classd elsew
	G30.0	Alzheimer's disease with early onset
	G30.1	Alzheimer's disease with late onset
	G30.8	Other Alzheimer's disease
	G30.9	Alzheimer's disease, unspecified
	G31.01	Picks disease
	G31.09	Other frontotemporal dementia
	G31.1	Senile degeneration
	G31.2	Degeneration of nervous system due to alcohol
	G94	Oth disorders of brain in diseases classd elsew
	R41.81	Age-related cognitive decline
	G31.83	Lewy body dementia

© 2023 Zissimopoulos J et al. *JAMA Network Open*
