# Supplementary material for: Trends in Incident Dementia Diagnosis Before and After Medicare Risk Adjustment
Source: JAMA Netw Open. 2023 Dec 15;6(12):e2347708. doi: 10.1001/jamanetworkopen.2023.47708 (PMC10724756; doi:10.1001/jamanetworkopen.2023.47708)
Supplement: Supplement 2. — Data Sharing Statement [file jamanetwopen-e2347708-s002.pdf]

Data Sharing Statement

Zissimopoulos. Patterns in Incident Dementia Diagnosis Before and After Medicare Risk Adjustment. *JAMA Netw Open*. Published December 15, 2023.
doi:10.1001/jamanetworkopen.2023.47708

Data
Data available: No

Additional Information
Explanation for why data not available: Medicare data is restricted use under DUA however we will share all code to replicate the study.
